# Alleviatory effects of Silicon on the morphology, physiology, and antioxidative mechanisms of wheat (*Triticum aestivum* L.) roots under cadmium stress in acidic nutrient solutions

**DOI:** 10.1038/s41598-020-80808-x

**Published:** 2021-01-21

**Authors:** Shafeeq ur Rahman, Qi Xuebin, Zhijuan Zhao, Zhenjie Du, Muhammad Imtiaz, Faisal Mehmood, Lu Hongfei, Babar Hussain, Muhammad Nadeem Ashraf

**Affiliations:** 1 Farmland Irrigation Research Institute, Chinese Academy of Agricultural Sciences, Xinxiang, 453003 China; 2 Key Laboratory of High-Efficient and Safe Utilization of Agriculture Water Resources of CAAS, Xinxiang, 453003 China; 3 Soil and Environmental Biotechnology Division, National Institute for Biotechnology and Genetic Engineering (NIBGE), Faisalabad, Pakistan; 4 Institute of Agricultural Resources and Regional Planning, Chinese Academy of Agricultural Sciences, Beijing, China

**Keywords:** Physiology, Plant sciences, Environmental sciences

## Abstract

Silicon (Si), as a quasi-essential element, has a vital role in alleviating the damaging effects of various environmental stresses on plants. Cadmium (Cd) stress is severe abiotic stress, especially in acidic ecological conditions, and Si can demolish the toxicity induced by Cd as well as acidic pH on plants. Based on these hypotheses, we demonstrated 2-repeated experiments to unfold the effects of Si as silica gel on the root morphology and physiology of wheat seedling under Cd as well as acidic stresses. For this purpose, we used nine treatments with three levels of Si nanoparticles (0, 1, and 3 mmol L^−1^) derived from sodium silicate (Na_2_SiO_3_) against three concentrations of Cd (0, 50, and 200 µmol L^−1^) in the form of cadmium chloride (CdCl_2_) with three replications were arranged in a complete randomized design. The pH of the nutrient solution was adjusted at 5. The averages of three random replications showed that the mutual impacts of Si and Cd in acidic pH on wheat roots depend on the concentrations of Si and Cd. The collective or particular influence of low or high levels of Si (1 or 3 mM) and acidic pH (5) improved the development of wheat roots, and the collective influence was more significant than that of a single parallel treatment. The combined effects of low or high concentrations of Cd (50 or 200 µM) and acidic pH significantly reduced root growth and biomass while increased antioxidants, and reactive oxygen species (ROS) contents. The incorporation of Si (1 or 3 mmol L^−1^) in Cd-contaminated acidic nutrient solution promoted the wheat root growth, decreased ROS contents, and further increased the antioxidants in the wheat roots compared with Cd single treatments in acidic pH. The demolishing effects were better with a high level of Si (3 mM) than the low level of Si (1 Mm). In conclusion, we could suggest Si as an effective beneficial nutrient that could participate actively in several morphological and physiological activities of roots in wheat plants grown under Cd and acidic pH stresses.

## Introduction

Cadmium (Cd), as a non-essential element, is a crucial hazardous pollutant in agricultural soils worldwide, mostly originated from sewage sludge, mining, industrial activities, or phosphate fertilizer application^1^. Generally, Cd absorbed in plants when cultivated in Cd-contaminated soils, which lead to retardation of growth, inhibition of photosynthesis, destruction of the antioxidative system, and nutrient imbalance, while potentially causing adverse impact on animal and human health if approaching the food-chain^2,3^. The high water solubility, relative mobility, and phytotoxicity of Cd make it a top priority pollutant for the researchers to develop various approaches to protect plants from Cd uptake, accumulation, and translocation.

Cd, like the other cations, follows the same apoplastic and symplastic pathways to move radially across the root layers^4^. Cd concentrations in the rhizospheric zone of plants inhibit root growth by enhancing root hairs near the root tip in radish (*Raphanus sativus* L.), and maize (*Zea mays* L.)^2^. A higher amount of Cd in the rhizosphere, however, resulted in decreased root hair development and the breakup of the 5,6 rhizodermis and external cortical cell layers^5,6^. The concentration of Cd in the shoot is largely determined by the entrance of Cd into the root, the sequestration in the root vacuoles, the displacement in xylem and phloem, and the suspension through the shoot^7^. Moreover, Cd concentration in roots is often but not always higher than in shoots^2^. Once Cd enters in plant body through roots, it produces reactive oxygen species (ROS) in a massive amount, which affects different physical and biochemical reactions in plants due to their oxidative capacity^8,9^. ROS in terms of hydroxyl radicals (OH^−^), hydrogen peroxide (H_2_O_2_), superoxide anions (O_2_^−^), and singlet oxygen (^1^O_2_), affects cell physiological pathways, membrane properties, signaling cascades, which ultimately cause cell death^10^.

Moreover, ROS causes DNA degradation, protein destruction, and lipid peroxidation in plants when produced in excessive amounts^11,12^. It has been established in grafting experiments that Cd concentrations in various plants are actively controlled by root properties^13,14^. To protect Cd translocation and accumulation in shoot tissues, it is urgent to adopt various strategies to improve the root morphology or develop some physical obstacles to the extracellular drive of Cd to ensure limited access of Cd ions to the xylem from the root apoplasm. For this purpose, we subjected the roots of wheat crop to Cd toxicity. Wheat (*Triticum aestivum* L.) is the second worldwide staple food after rice for a broad sector of people complete 20% daily protein need of 4.5 billion people all over the world^15^. It can adopt a broad range of environmental and soil conditions; however, a significant decline in its growth and production is being caused by heavy metal-contaminated soils, especially Cd^16^.

The results of various studies have established soil pH as an essential and dominant soil property for regulating sorption/desorption and solubility of supplementary Cd or natural soil Cd toxicity^17,18^, therefore expected to play a critical role inducing Cd phytoavailability. Soil pH due to comparatively easy to operate may be helpful to control and management of plant Cd accumulation. Also, the Cd uptake and absorption capacity of crops are strongly correlated with their species or cultivars^13,19,20^. However, while several scientists conducted numerous studies to create a link between soil pH and plant uptake of Cd, the majority of these were conducted using soils to which soluble inorganic Cd salts were added^18^, or using soils Cd contaminated from sewage sludge^21^. In the present study, we arranged a hydroponic study to address the effect of silicon (Si) on Cd uptake and accumulation in an acidic nutrient solution in the wheat plants without the influence of other soil-related factors.

To overcome the adverse effects of Cd toxicity, various methods have been adopted by previous researchers, but the most economical, environment-friendly, and defensible approach is the supply of quasi-essential to plants^22,23^. In this regard, silicon (Si) has been proved a suitable candidate to demolish biotic and abiotic stresses, as well as Cd toxicity in previous studies^24–26^. However, up to date, various studies have been carried out in wheat plants as for the advantageous effects of Si on Cd stress mitigation, which developed a high demand for this element in the present era. The novelty of the present study is that we conducted an experiment to see the protective role of Si on root morphological and physiological traits of wheat plants grown under the Cd-contaminated acidic nutrient solution.

Being the second most sufficient element on the earth's crust, silicon (Si) mitigates numerous forms of biotic and abiotic stresses^27–29^. High silica activates physical and biochemical defense mechanisms in plant tissues to increase stress tolerance, specifically in graminaceous plants such as maize, barley, rice, and wheat^30–33^. Si plays a crucial role in relieving heavy metal stress by various mechanisms, including decreased Cd uptake and displacement from roots to shoots, thus preventing Cd's harmful impact on photosynthetic machinery and grains^34,35^. However, Si traps a high concentration of Cd in roots by vacuolar sequestrations^34^, resulting in a reduction of Cd translocation in plant aerial parts^36^. Liang, et al.^37^ stated that Si co-precipitates with Cd, subsequently strong binding of Cd to the cell wall and the cytosol or symplast accumulation of Cd. Numerous studies have demonstrated Si's beneficial effects on plant growth and enlargement, photosynthetic machinery, balanced nutrient availability, and mechanisms for ROS scavenging^29,31^.

To our best knowledge, there is less literature available to address the beneficial role of Si in terms of root morphology and physiology of wheat plants against Cd toxicity under acidic environmental conditions. Therefore, the objective of these 2-repeated hydroponic studies was to explore the role and mechanism of Si to alleviate Cd toxicity in terms of Cd uptake and accumulation in roots, root morphological and physiological characteristic, anti-oxidants in root organs, and ROS (hydrogen peroxide; H_2_O_2_) production, lipid peroxidation in terms of MDA contents in roots in Cd-stressed-wheat plants. We also assessed the role of Si in the availability of macro and microelements in roots of wheat crops. The same experiment was installed twice at the same experimental site to reduce potential errors. Three replications out of six were selected randomly to finalize our results.

## Materials and methods

### Plant culture and experimental design

“The present studies were conducted at the experimental site of Farmland Irrigation Research Institute, Chinese Academy of Agricultural Sciences, Xinxiang, China”^8^. All research had adopted the same preliminary model. At the end of the experiments, the three replications out of six were randomly chosen to finalize the tests. The seed of the same wheat genotype (Xin Mai 23) was used in both studies. Healthy seeds were soaked in double distilled water overnight and then sown in filtered trays of quartz clay. These sand trays were mounted in a control room with a light intensity of 370 μmole m^−2^ S^−1^, and the photoperiod was 16 h/8 h (day/dark). The control room temperature was set to 27 °C to 30 °C and retained relative humidity of 86 percent. At root shoot junction, the 2 weeks old seedlings were enfolded with foam and set in 15 in. × 17 in. size holes of 10 L water volume plastic sheets floating on a plastic tub. These plastic containers were filled with a solution of 3/4L Hoagland's solution^38^ (See Online appendix heading 1). The nutrient solution was replaced every 3 days interval.

The 1-week-old wheat seedlings were transplanted in half-strength Hoagland's solution. The Hoagland solution was exchanged with full-strength after 20 days of transplantation till the end of the experiment, and the pH of the growing media was modified to 5 (acidic) with 81% phosphoric acid using a PHS-29A pH meter. The treatments were given after 20 days of transplanting for 3 weeks (21 days). Silicon (Si) nanoparticles (See Online appendix heading 2) made by sodium silicate (Na_2_SiO_3_) was added with the rate of 0, 1, and 3 mmol L^−1^ while, Cadmium (Cd) in the form of cadmium chloride (CdCl_2_) was supplemented with the rate of 0, 50 and 200 µmol L^−1^ in the nutrient solution of acidic pH (5). Si treatments were introduced when seedlings were 27 days old; the 0, 1, and 3 mmol L^−1^ Si solutions with neutral pH were attained by mixing a sufficient amount of Na_2_SiO_3_ in the double-distilled water. The acidic pH (5) was maintained three times a day with 0.1 M HCl using a PHS-29A pH meter. After every 3 days, the Hoagland solution was renewed to sustain the pH value, and double-distilled water was added to keep the volume of the container. The treatments were also re-introduced with the renewal of nutrient solution.

The pots were then distributed into nine treatments/groups each of 3 pots. In group one plant were exposed at Cd 50 µmol L^−1^ and not received any concentration of Si. In second and third groups of pots, plants were exposed at Cd 50 along with Si 1 and 3 mmol L^−1^ in the Hoagland solution, respectively. In group 4, pots were exposed at Cd 200 µmol L^−1^ and not received any concentration of Si. In group 5 and 6 pots were exposed at Cd 200 µmol L^−1^ along with Si 1 and 3 mmol L^−1^ in Hoagland solution, respectively. In group 7, pots were exposed at Si 1 mmol L^−1^ and not received any concentration of Cd. In group 8, pots were exposed at Si 3 mmol L^−1^ and not received any concentration of Cd. In a group, nine pots were exposed at Hoagland solution and not received any concentration of Cd and Si (control). Three replications for each treatment were used in both experiments. The pots were randomly placed inside the control room. Both experiments were performed in natural conditions at an ambient temperature of 22 to 30 °C during daytime and 15–20 °C during nighttime. After 120 days of transplantation, all the plants were sampled. Root samples for extraction of the enzyme assays were immediately frozen in liquid nitrogen and stored at − 80 °C.

### Determination of root biomass and root morphological traits

Plants were sampled after 120 days of germination for assessment of root biomass and root morphological traits, including root tips, average root diameter, root volume, and root length. Root fresh weight was measured immediately after harvesting the plants' samples. “Took two root samples from each replication and placed in an oven at 105 C for 30 min, and then kept at 80ºC for 24 h to get constant weight and measured the dry weight”^26^. Remaining root parameters such as root width, average root diameter, root tips, and root length were measured using a root automated scan tool (MIN MAC, STD I600), fitted with a 4.1 Win RHIZO (Arsenault et al., 1995) commercial software package provided by Regent Instruments, 2000 Business, USA.

### Biochemical analysis

“Enzymatic antioxidants (superoxide dismutase: SOD, catalase: CAT, guaiacol peroxidase: POD), non-enzymatic antioxidants (proline), lipid peroxidation contents in terms of malondialdehyde (MDA), and reactive oxygen species in term of hydrogen peroxide (H_2_O_2_) in three random replications of root samples of wheat plants were assessed by using the kits of Beijing Solarbio Science & Technology Co., Ltd ((http://www.solarbio.com). Briefly, 0.5 g fresh samples of roots were milled with the help of a motor and pestle and standardized in 0.05 M phosphate buffer with pH 7.8 under chilled condition. The standardized mixture was centrifuged at 12,000 rpm for 10 min at 4 °C after sieving through four layers of muslin cloth. The activity of CAT was assessed by the following formula:

1 $$CAT\left( {\frac{\upmu }{{{\text{mgprot}}}}} \right) = \left( {ODControl - ODTest} \right) \times \frac{271}{{60}} \times \frac{1}{S Q} \times \frac{1}{Protein conc. }$$

SQ = Sample Quantity, OD_control_ = absorption of light in control, OD_test_ = absorption of light in test samples.

After mixing all reagents in the standardized mixture, the supernatant was again centrifuged at 3500 rpm for 10 min. The light diameter of 1 cm was adjusted to zero by double streaming water. OD was measured at 420 nm wavelength. The activity of POD was measured by the following equation:

2 $$POD\left( {\frac{\upmu }{{{\text{mgprot}}}}} \right) = \left( {ODTest - ODControl} \right) \times \frac{12}{{1\;{\text{cm}}}} \times \frac{Vt}{{S Q \times RT \times Protein conc. }} \times 1000$$

V_t_ = Total volume of the reaction liquid, SQ = Sample Quantity, RT = Reaction time, OD_control_ = absorption of light in control, OD_test_ = absorption of light in test samples.

After mixing all reagents in a standardized mixture, the supernatant was placed at room temperature for 10 min. SOD was measured at 550 nm wavelength. The activity of SOD was measured by the following equation:

3 $$SOD\left( {\frac{\upmu }{{{\text{mgprot}}}}} \right) = \left( {\frac{ODcontrol - ODtest}{{ODControl}}} \right) \times \frac{1}{50} \times \frac{Vt}{{S Q \times Protein conc. }}$$

V_t_ = Total volume of the reaction liquid, SQ = Sample Quantity, OD_control_ = absorption of light in control, OD_test_ = absorption of light in test samples.

The level of lipid peroxidation in the leaf tissue was assessed by measuring the contents of malondialdehyde (MDA), a by-product of lipid peroxidation. Briefly, 0.2–0.5 g weighted fresh samples of roots were milled with the help of a motor and added 2 ml 10% TCA and a small amount of quartz sand, ground to homogenate, add 3 ml TCA, further ground. The homogenized sample was centrifuged at 12,000 rpm for 10 min. Took 2 ml supernatant, added 0.67% TBA, mixed and boiled for 15 min in 100 ºC water bath. Cooled the sample at room temperature and centrifuged again. Absorption values of samples were measured at 532 nm, 600 nm, and 450 nm, respectively. The activity of MDA was measured by the following formula:

4 $$CMDA = 6.45\left( {A532 - A600} \right) - 0.56 \times A450$$

5 $$MDA\left( {\frac{{\upmu {\text{mol}}}}{{\text{g}}}} \right) = CMDA \times \left( {\frac{Vt}{{S Q \times 1000}}} \right)$$

V_t_ = Total volume of the reaction liquid, SQ = Sample Quantity.

Proline was also assessed by using the kit of Beijing Solarbio Science & Technology Co., Ltd. The following formula was used to measure the proline contents:

6 $$Proline\left( {\frac{{\upmu {\text{g}}}}{{\text{g}}}} \right) = \left( {\frac{ODsample - ODblank}{{ODst - ODblank}}} \right) \times Cst\frac{{5\,\upmu {\text{g}}}}{{{\text{ml}}}} \times \frac{Vreagent}{{Mtissue }} \times COD$$

where CoD = the coefficient of dilution in the pre-treatment process.

Similarly, H_2_O_2_ was also assessed by using the kit of Beijing Solarbio Science & Technology Co., Ltd. The following formula was used to measure the H_2_O_2_ contents**:**

7 $$H_{2} O_{2} \left( {\upmu {\text{M}}} \right) = \left( {\frac{ODsample - ODblank}{{ODst - ODblank}}} \right) \times Cst163\;\upmu {\text{M}} \times COD$$

CoD = the coefficient of dilution in the pre-treatment process, C_st_ = Concentration of standard, OD_st_ = Absorption of standard sample”^8^.

Ascorbic acid (AsA) contents were measured by the method of Mukherjee and Choudhuri^39^. We measurement AsA contents in the extract of trichloroacetic acid combined with dinitrophenylhydrazine and thiourea. Subsequently, the mixture was boiled for 15 min in a water bath (HWS-28), cooled at 27ºC, added H_2_SO_4,_ and absorbance was measured at 530 nm with the help of a spectrophotometer (TU-1810).

Meanwhile, glutathione (GHS) concentration in roots was assessed by the technique of Griffith^40^. For the measurement of GSH first, we neutralized metaphosphoric acid with sodium citrate. Analysis of three replicates of each treatment we made 700 μl of 0.3 mM NADPH, 100 μl of 6 mM 5, 5′-dithiol-bis-2-nitro benzoic acid, and 100 μl distilled water and100 μl of extract. In the end, GSH reductase (10 μl of 50 Units ml^‒1^) was added, and a spectrophotometer (TU-1810) was used to detect the absorbance at 412 nm to calculate GSH contents from a standard arc.

Relative water contents (RWC) in the roots of wheat plants were assessed by using the method of WEATHERLEY^41^ with some modification of Osman and Rady^42^. Similarly, the method of Premachandra et al.^43^ with some modifications by Rady, et al.^16^ was used to measure membrane stability index (MSI).

The method of Irigoyen, et al.^44^ was used to measure total soluble sugar (TSS) contents in oven-dried roots of wheat plants. Took 0.2 g of dried root sample and homogenized with 5 ml 96% (v/v) ethanol, and then eroded with 5 ml 70% (v/v) ethanol. The extract was centrifuged with a centrifuge (TGL-18 M) at 3500 × g for 10 min, and then stored the supernatant at 4 °C. The water bath (HWS-28) was used to boil the 0.1 ml extract of ethanol along with 3 ml anthrone reagent [150 mg anthrone + 100 ml of sulphuric acid; 72%, v/v] for 10 min. The absorption was measured with a spectrophotometer at 625 nm, after cooling the supernatant at room temperature.

### Determination of Cd, Si, and nutrient elements in plant tissues

“The contents of micronutrients (nitrogen: N, phosphorus: P, and potassium: K), macronutrients (calcium: Ca, magnesium: Mg, and zinc: Zn), heavy metal Cd contents, and beneficial elements Si contents were assessed by inductively coupled plasma mass spectroscopy (ICP-MS, Agilent, and 7700 X, USA) after being oven-dried by following the methods of previous researchers^8^ (See Online appendix heading 3 and 4).

### Statistical analysis

“The data were processed and analyzed using the SPSS 21.0 (SPSS, Chicago, IL), and all the graphs were made using the Sigma plot 12.5 software packages. The means of the three random replicates of 2-repeated experiments were subjected to analysis of variance (ANOVA), and multiple comparisons were performed using Duncan's multiple range test (DMR) at *P* < 0.05^8^.

## Results

### Effect of silicon and cadmium on root biomass

**Figure 1:**
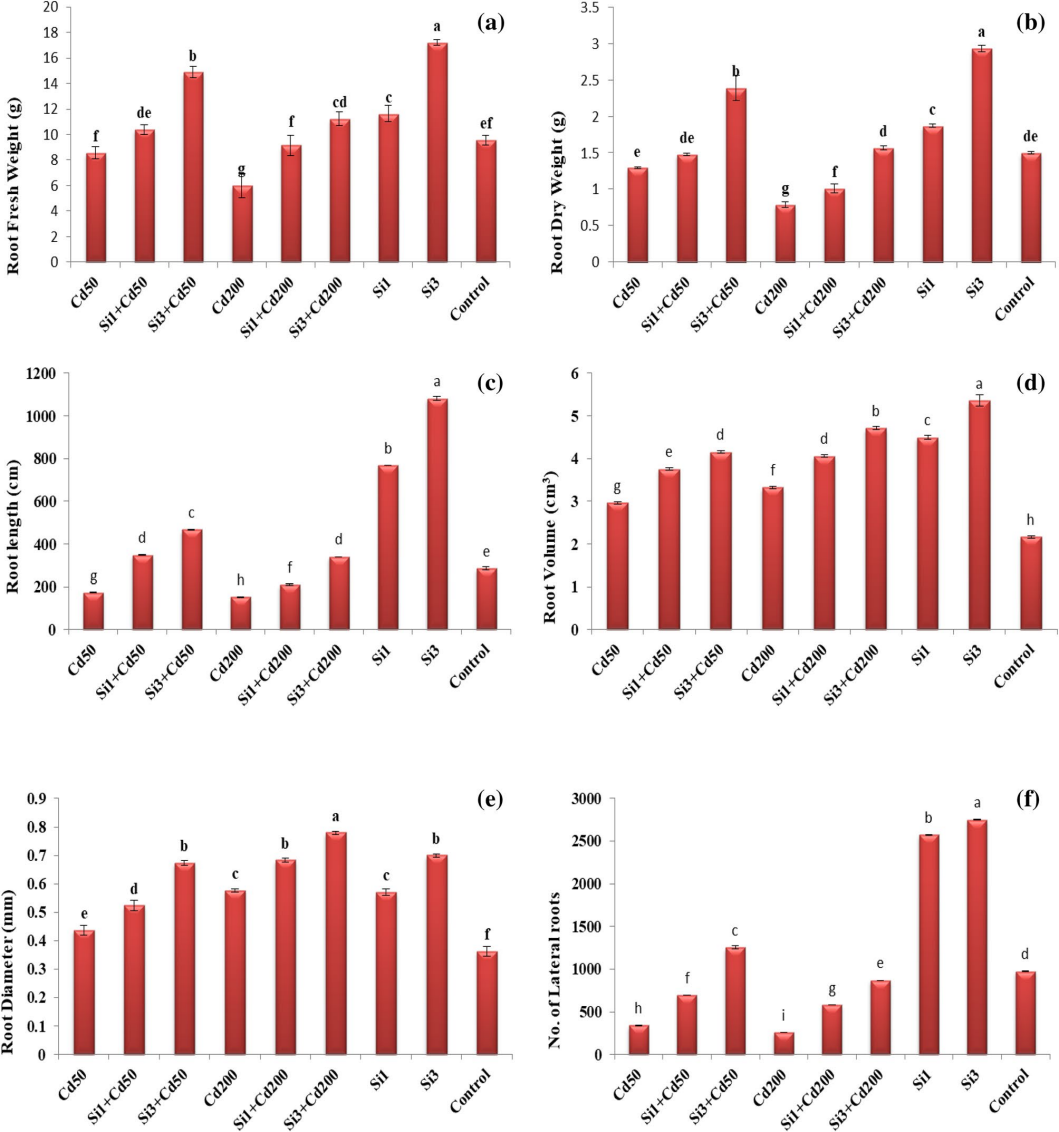
Effect of Si and Cd on root fresh weight (RFW) (g plant^−1^), root dry weight (RDW) (g plant^−1^), root length (R.L.) (cm), root diameter (R.D.) (mm), root volume (R.V.) (cm^3^), root tips (R.T.) (no. of lateral roots) contents in the roots of wheat plants. The values are expressed as the mean ± S.D. (n = 3). The different superscript letters within a column indicate significant differences at *P* < 0.05.

The effect of silicon (Si) and cadmium (Cd) on root fresh and dry weight of wheat plants grown under acidic nutrient solution is shown in Fig. 1. The means of three random replicates of 2-repeated experiments showed that Cd concentrations in acidic nutrient solution significantly reduced root fresh and dry weight as compared to the control. Root fresh weight for Cd50 and Cd200 was decreased by 10% and 37%, respectively, than that of control. Similarly, root dry weight for Cd 50 and 200 µmol L^−1^ was reduced by 14% and 47%, respectively, than that of control. The application of Si in Cd contaminated acidic nutrient solution significantly alleviated Cd stress by increasing root fresh and dry weight (Fig. 1). Si application with 1 and 3 mmol L^−1^ along with Cd 50 and 200 µmol L^–1^ increased fresh root weight by 21%, 73%, and 52%, 87%, respectively, as parallel to alone Cd 50 and Cd 200. Similarly, Si 1 and 3 mmol L^−1^ along with Cd50 and Cd200 increased root dry weight by 14%, 84%, and 28%, 98%, respectively, as parallel to alone Cd50 and Cd200.

### Effect of silicon and cadmium on root volume and average diameter

The effect of Cd and Si on root volume and diameter is shown in Fig. 1. The means of three random replicates of 2-repeated experiments showed that all levels of Cd in an acidic nutrient environment significantly increased root volume and diameter than that of control. Cd with the concentration of 50 and 200 µmol L^−1^ increased root volume by 37% and 54% and increased root diameter by 20% and 59%, respectively, than that of control (acidic pH). The addition of Si in Cd-contaminated acidic solution significantly alleviated Cd toxicity by further increasing root volume and diameter (Fig. 1). Si 1 mmol L^−1^, along with Cd50 and Cd200, significantly increased root volume by 27% and 22% and increased root diameter by 20% and 18%, respectively, as parallel to alone Cd50 and Cd200.

Similarly, Si 3 mmol L^−1^, along with Cd50 and Cd200, significantly increased root volume by 40% and 42% and increased root diameter by 54% and 35%, respectively, as compared with Cd50 and Cd200. The most significant results were recorded with Si 3 mmol L^−1^ as paralleled to Si 1 mmol L^−1^ concentration against the same levels of Cd. Moreover, Si single application with a preference of 3 mmol L^−1^ in an acidic solution also led to a significant increase in root volume and average diameter than that of control (Fig. 1).

### Effect of silicon and cadmium on total root length and root tips

The effects of Si and Cd on wheat plants in terms of root length and root tips are shown in Fig. 1. The means of three random replicates of 2-repeated experiments showed that all levels of Cd concentration in an acidic nutrient solution significantly decreased root length and root tips than that of control (Fig. 1). Cd concentration with 50 and 200 µmol La^−1^ significantly reduced root length by 39% and 47% and decreased root tips by 65% and 73%, respectively, than that of control (acidic pH 5). The addition of Si in Cd-contaminated acidic nutrient solution significantly encountered the adverse effects of both Cd and acidic stress by elevating root length and root tips. Si 1 mmol L^−1^, along with Cd50 and Cd200, significantly increased root length by 101% and 38%, and increased root tips by 105% and 126%, respectively, as paralleled to alone Cd50 and Cd200. Similarly, Si 3 mmol L^−1^, along with Cd50 and Cd200, increased root length by 169% and 124%, and increased root tips by 270% and 237%, respectively, as paralleled with alone Cd50 and Cd200. Moreover, Si single application with a preference of 3 mmol L^−1^ in an acidic solution also led to a significant increase in root length and root tips as paralleled to control (Fig. 1).

### Effect of silicon and cadmium on enzymatic antioxidant and protein contents

The effect of Cd and Si on enzymatic (catalase; CAT, superoxide dismutase; SOD, peroxidase; POD) antioxidants in the roots of wheat plants grown in the acidic nutrient solution are shown in Table 1. The means of three random replicates of 2 back to back experiments showed that all levels of Cd concentrations (50 and 200 µmol L^−1^) significantly increased the contents of antioxidants in the roots of wheat plants as paralleled to control (Table 1). Cd with the concentrations of 50 and 200 µmol L^−1^ significantly increased CAT by 52% and 112%, SOD by 22% and 32%, and POD by 51% and 139%, respectively, in the roots of wheat plants as paralleled to control. The addition of Si with the preference of 3 mmol L^−1^ in Cd-contaminated acidic solution significantly encountered both Cd and acidic toxicities by further elevating enzymatic antioxidant contents in roots of wheat plants. Si 3 mmol L^−1^ along with Cd50 and Cd200 significantly increased CAT contents by 145% and 277%, SOD contents by 60% and 61%, and POD contents by 89% and 63%, respectively as paralleled to alone Cd50 and Cd200. Moreover, Si alone application in highly acidic nutrient solution also led to significant increased enzymatic antioxidants in the roots of wheat plants (Table 1).

**Table 1:** Effect of three different levels of Si (0, 1 and 3 mmol L^−1^) on protein, enzymatic and non-enzymatic antioxidants and reactive oxygen species of cadmium (50 and 200 µmol L^–1^)-stressed wheat plants.

Treatment	CAT (unit mg^−1^ protein)	SOD (unit mg^−1^ protein)	POD (unit mg^−1^ protein)	Protein (ug g^−1^)	AsA (µmol g^‒1^ DW)	GHS (µmol g^‒1^ DW)	TSS (mg g^‒1^ DW)	Proline (µg g^−1^FW)	MDA (µmol mg^−1^ FW)	H_2_O_2_ (µmol g^−1^ FW)
Cd50	0.54 ± 0.02ef	11.32 ± 0.24e	6.55 ± 0.18g	4.59 ± 0.08g	1.66 ± 0.02f	0.31 ± 0.003d	24.11 ± 0.31f	0.06 ± 0.001e	15.67 ± 0.24c	63.83 ± 1.35b
Si1 + Cd50	0.86 ± 0.03d	12.81 ± 0.32d	9.44 ± 0.21e	7.55 ± 0.07e	2.40 ± 0.04d	0.40 ± 0.005c	31.11 ± 0.15e	0.09 ± 0.002c	9.41 ± 0.28e	46.91 ± 1.35d
Si3 + Cd50	1.33 ± 0.19c	18.12 ± 0.40b	12.38 ± 0.27c	14.53 ± 0.27b	3.03 ± 0.04b	0.48 ± 0.006b	53.40 ± 1.05a	0.10 ± 0.003b	7.34 ± 0.20f	21.65 ± 0.32f
Cd200	0.76 ± 0.02de	12.25 ± 0.23de	10.36 ± 0.36d	1.65 ± 0.17h	2.04 ± 0.04e	0.39 ± 0.011c	33.54 ± 0.27d	0.07 ± 0.006c	29.61 ± 0.36a	90.32 ± 0.47a
Si1 + Cd200	1.27 ± 0.05c	15.08 ± 0.37c	14.86 ± 0.15b	7.92 ± 0.28e	2.84 ± 0.04c	0.49 ± 0.009b	38.01 ± 0.12c	0.10 ± 0.001b	20.48 ± 0.43b	53.98 ± 0.82c
Si3 + Cd200	2.85 ± 0.08a	19.66 ± 0.30a	16.85 ± 0.27a	12.31 ± 0.12c	3.67 ± 0.01a	0.58 ± 0.007a	44.74 ± 0.22b	0.13 ± 0.007a	15.76 ± 0.12c	45.08 ± 0.53e
Si1	1.76 ± 0.03b	12.45 ± 0.14d	8.46 ± 0.30f	11.61 ± 0.23d	1.36 ± 0.01g	0.30 ± 0.006d	19.31 ± 0.31g	0.07 ± 0.001c	6.78 ± 0.04f	16.58 ± 0.47g
Si3	1.93 ± 0.02b	14.73 ± 0.10c	10.74 ± 0.32d	18.16 ± 0.26a	1.96 ± 0.02e	0.40 ± 0.007c	23.21 ± 0.10f	0.09 ± 0.016d	4.62 ± 0.15g	11.13 ± 0.48h
Control	0.36 ± 0.02f	9.29 ± 0.68f	4.33 ± 0.6h	6.47 ± 0.07f	0.99 ± 0.01h	0.21 ± 0.003e	12.56 ± 0.09h	0.05 ± 0.001f	10.75 ± 0.39g	41.78 ± 1.25d

Where CAT, SOD, POD, GHS, TSS, AsA, MDA, and H_2_O_2_ stand for catalase, superoxide dismutase, peroxidase, glutathione, total soluble sugar, ascorbic acid, malondialdehyde, and hydrogen peroxide, respectively. Means ± S.D. (n = 3) with different letters in the column indicates significant (*P* ≤ 0.005) differences between treatments.

The estimation of protein contents also indicated that Si treatments significantly effect on the roots of wheat plants with and without Cd stress (Table 1). The recorded data showed that protein contents in roots increased with the decreased in Cd concentration from 200 to 50 µmol L^–1^. The maximum protein contents were observed when plants were treated with 3 mmol L^−1^ Si alone, while minimum protein contents were observed when plants were grown under 200 µM toxicity of Cd. As paralleled to control and Cd-treated-plants, soluble protein contents were highest in both levels of Si (1 and 3 mmol L^−1^). Similarly, protein contents in Si, along with Cd treated plants, were also greater as paralleled to Cd alone (50 and 200 µmol L^−1^) (Table 1).

### Effect of silicon and cadmium on non-enzymatic antioxidants

The effect of Cd and Si on non-enzymatic (ascorbic acid; AsA and glutathione; GHS) antioxidants in wheat roots grown in the acidic growing medium, as shown in Table 1. The means of three random replicates of 2-repeated experiments showed that all levels of Cd concentrations (50 and 200 µmol L^–1^) significantly increased the non-enzymatic antioxidants contents in the roots of wheat plants as paralleled to control (Table 1). Cd with the concentrations of 50 and 200 µmol L^−1^ significantly increased AsA by 67% and 106%, and GHS by 44% and 83%, respectively, in the roots of wheat plants as related to control. The application of Si with the preference of 3 mmol L^–1^ in Cd-contaminated acidic solution significantly encountered both Cd and acidic toxicities by further elevating non-enzymatic antioxidant contents in roots of wheat plants. Si 3 mmol L^−1^ along with Cd50 and Cd200 significantly increased AsA contents by 83% and 80% and GHS contents by 55% and 48%, respectively, as related to alone Cd50 and Cd200. Moreover, Si alone application in highly acidic nutrient solution also led to significant increased non-enzymatic antioxidants in the roots of wheat plants (Table 1).

### Effect of silicon and cadmium on osmoprotectants

The effect of Cd and Si on osmoprotectants (total soluble sugars; TSS and proline) in the wheat roots grown in the acidic nutrient solution is shown in Table 1. The means of three random replicates of two back to back experiments revealed that all levels of Cd concentrations (50 and 200 µmol L^–1^) increased the contents of osmoprotectants in the roots of wheat plants as related to control (Table 1). Cd with the concentrations of 50 and 200 µmol L^−1^ significantly increased TSS by 92% and 167%, and proline by 19% and 66%, respectively, in the roots of wheat plants as related to control. The application of Si with the preference of 3 mmol L^−1^ in Cd-contaminated acidic solution significantly encountered both Cd and acidic toxicities by further elevating osmoprotectants contents in roots of wheat plants. Si 3 mmol L^−1^, along with Cd50 and Cd200, significantly increased TSS contents by 121% and 33% and proline contents by 82% and 62%, respectively, as related to alone Cd50 and Cd200. Moreover, Si alone application in highly acidic nutrient solution also leads to increase osmoprotectants in the roots of wheat plants (Table 1).

### Effect of silicon and cadmium on reactive oxygen species (ROS) and malondialdehyde contents

The effect of Cd and Si on ROS in terms of hydrogen peroxide (H_2_O_2_) and lipid peroxidation in terms of malondialdehyde (MDA) contents in the roots of wheat plants grown in the acidic growing media is shown in Table 1. The means of three random replicates of two repeated experiments displayed that all levels of Cd concentrations (50 and 200 µmol L^–1^) remarkably increased the contents of ROS in the wheat roots as related to control (Table 1). Cd with the concentrations of 50 and 200 µmol L^–1^ significantly increased MDA by 46% and 175%, and H_2_O_2_ by 53% and 116%, respectively, in the wheat roots as paralleled to control. The application of Si with the preference of 3 mmol L^−1^ in Cd-contaminated acidic solution significantly encountered both Cd and acidic toxicities by decreasing ROS contents in roots of wheat plants. Si 3 mmol L^−1^, along with Cd50 and Cd200, significantly decreased MDA contents by 53% and 47% and H_2_O_2_ contents by 66% and 50%, respectively, as related to alone Cd50 and Cd200. Moreover, Si alone application in highly acidic nutrient solution also led to remarkably reduced H_2_O_2_ and MDA contents in the roots of wheat plants (Table 1).

### Tissue-specific silicon concentration in roots

The concentration of Si in the roots of wheat plants with and without Cd exposure in the acidic nutrient solution is shown in Table 2. The means of three random replicates of two back to back experiments showed that Si concentration was increased in root tissues with the rise of Cd level in the acidic growing media. There was an antagonistic correlation with the concentration of Cd and the accumulation of Si in the wheat roots. Si concentration in Si1 + Cd200 was 17% higher as compared with Si1 + Cd50. Similarly, Si concentration in Si3 + Cd200 was 26% higher as compared with Si3 + Cd50 (Table 2).

**Table 2:** Effect of Si and Cd on nitrogen (N) (mg g^−1^), phosphorus (P) (mg g^−1^), potassium (K) (mg g^−1^), calcium (Ca) (g kg^−1^), magnesium (Mg) (g kg^−1^), zinc (Zn) (mg kg^−1^), cadmium (Cd) (mg kg^−1^), and silicon (Si) (mg kg^−1^), contents in the roots of wheat plants.

Treatments	N (mg g^−1^)	P (mg g^−1^)	K (mg g^−1^)	Ca (g kg^−1^)	Mg (g kg^−1^)	Zn (mg kg^−1^)	Cd (mg kg^−1^)	Si (mg kg^−1^)
Cd50	24.38 ± 0.53g	2.66 ± 0.03f	11.93 ± 0.34g	9.68 ± 0.25f	1.05 ± 0.006f	38.64 ± 5.65e	1361.82 ± 2.63d	0.06 ± 0.01g
Si1 + Cd50	32.71 ± 0.35d	3.27 ± 0.08e	15.64 ± 0.18e	13.46 ± 0.23c	1.15 ± 0.004e	48.02 ± 12.54c	1154.34 ± 8.92e	160.75 ± 0.53e
Si3 + Cd50	36.49 ± 0.30c	3.97 ± 0.03d	19.27 ± 0.33c	15.73 ± 0.04b	1.26 ± 0.015d	63.93 ± 8.83b	437.62 ± 5.10f	208.25 ± 6.31c
Cd200	18.61 ± 0.35h	1.11 ± 0.01g	8.37 ± 0.18h	7.69 ± 0.16g	0.85 ± 0.005g	18.45 ± 2.13g	2773.11 ± 7.79a	0.06 ± 0.02g
Si1 + Cd200	25.88 ± 0.37f	2.73 ± 0.09f	11.98 ± 0.04g	9.92 ± 0.05f	1.07 ± 0.010f	28.83 ± 5.27f	2110.88 ± 12.26b	187.56 ± 2.05d
Si3 + Cd200	30.94 ± 0.03e	3.38 ± 0.18e	14.69 ± 0.17f	12.68 ± 0.18d	1.36 ± 0.004c	47.57 ± 7.46c	1646.68 ± 6.51c	283.31 ± 2.71a
Si1	38.93 ± 0.55b	5.30 ± 0.12b	20.80 ± 0.21b	13.68 ± 0.04c	1.56 ± 0.005b	65.24 ± 7.52b	0.07 ± 0.00g	107.75 ± 3.39f
Si3	45.17 ± 0.19a	6.22 ± 0.06a	25.32 ± 0.21a	16.77 ± 0.13a	1.78 ± 0.007a	75.76 ± 12.48a	0.08 ± 0.01g	242.78 ± 2.46b
Control	31.32 ± 0.50e	4.33 ± 0.19c	18.23 ± 0.62d	11.51 ± 0.28e	1.24 ± 0.019d	42.27 ± 7.91d	0.07 ± 0.01g	0.01 ± 0.00g

The values are expressed as the mean ± S.D. (n = 3). The different superscript letters within a column indicate significant differences at *P* < 0.005.

### Tissue-specific cadmium concentration in roots

Cd accumulation in roots of wheat plants with and without Si application is shown in Table 2. Without Si application, Cd accumulation in roots was enhanced with the rise of the Cd level from 50 to 200 µmol L^−1^. While Si (1 and 3 mmol L^−1^) addition in acidic nutrient solution significantly hindered Cd uptake, translocate, and accumulation in wheat plants. But most significant results were recorded at Si 3 mmol L^−1^ compared to Si 1 mmol L^−1^ against both levels of Cd (50 and 200 µM). Si at the concentration of 1 mmol L^–1^ along with Cd50 and Cd200 reduced Cd accumulation by 15% and 23% in roots as compared to alone Cd50 and Cd200. Similarly, Si at the level of 3 mmol L^−1^ along with Cd50 and Cd200 reduced Cd accumulation by 68% and 41% in roots as parallel to alone Cd50 and Cd200 (Table 2).

### Effect of silicon and cadmium on nutrient concentrations in roots of wheat plants

The influence of Cd and Si on the accumulations of macro and microelements in the roots of wheat plants grown in acidic nutrient solution are shown in Table 2. The means of three random replicates of 2-repeated experiments revealed that all levels of Cd (50 and 200 µmol L^−1^) significantly decreased the concentration of N, P, K, Ca, Mg, and Zn in the roots of wheat plants. Cd with the concentration of 50 and 200 µmol L^–1^ significantly decreased N concentrations by 46% and 59%, P concentrations by 38% and 74%, K concentration by 34% and 54%, Ca concentrations by 16% and 33%, Mg concentrations by 16% and 31%, and Zn concentrations by 8% and 56%, respectively in the roots of wheat plants as parallel to control (Table 2). The addition of Si with the preference of 3 mmol L^−1^ in Cd-contaminated acidic solution significantly reversed the toxicity of both Cd and acidic pH toxicities by elevating the concentrations of all essential nutrients. Si 3 mmol L^−1^ along with Cd50 and Cd200 significantly increased N concentrations by 50% and 66%, P concentrations by 49% and 205%, K concentrations by 61% and 75%, Ca concentrations by 63% and 65%, Mg concentrations by 21% and 59%, and Zn concentrations by 65% and 158%, respectively as parallel to Cd50 and Cd200. Si single application with a preference of 3 mmol L^−1^ in an acidic solution also led to a significant increase in concentrations of all recorded essential nutrients as parallel to control (Table 2).

## Discussion

The root physiological and morphological traits display significant alterations in response to various environmental stresses^45–47^, and our findings showed the influence of Cd and Si on roots morphology and physiology of wheat crop grown in acidic nutrient solutions. First, the roots of wheat crops treated with moderate and high levels of Cd (50 or 200 µmol L^−1^) along with acidic pH (5) showed significant decreased growth and development of roots against control (acidic pH). Notably, the root morphological and physiological traits of wheat seedlings simultaneously treated with a low or moderate concentration of Si (1 and 3 mM) and low or high levels of Cd (50 and 200 µmol L^−1^) in acidic environmental conditions were enhanced than that of wheat seedlings treated to the parallel Cd and low pH particular treatments. Second, treatment with a small and moderate concentration of Si (1 and 3 mmol L^−1^) had positive effects on root morphology and physiology as compared with low pH single treatment (Fig. 1); besides, the biomass and phenotype of roots of wheat seedlings exposed under high concentration of Si (3 mmol L^−1^) along with acidic pH were significantly enhanced than that of roots treated with Si low concentration (1 mM) and the roots treated with both levels of Cd (50 and 200 µmol L^−1^) along with low pH. These findings showed that the inhibitory effects of acidic pH and low and high concentrations of Cd (50 and 200 µM) on the root morphology and physiology were alleviated by the treatment of Si (Fig. 1). Third, the effect of Si application on root morphology and physiology strongly correlated with the concentration of Si (Fig. 1), and the two way ANOVA results shown an apparent collaboration among Si and Cd concentrations along with acidic pH (Fig. 1 and Table 1).

Root growth and development directly influence the root morphology and physiology. Our study demonstrated that the root physiology and morphology treated with Cd along with acidic pH is negatively correlated with the biomass of the root (Fig. 1). Our research is the consistency of previous findings where Cd concentration inhibited 50% root growth by increasing root hairs near the root tip in numerous plant species^5,48^. Moreover, previous findings have shown that Cd concentrations increased root diameter without causing necrosis^49,50^. Our study demonstrated the same results where low and high concentrations of Cd initiated a remarkable increase in average root diameter and root volume. At the same time, decrease average root biomass, root length, and the number of lateral roots (Fig. 1). The increased diameter and volume of Cd-treated root of wheat seedlings might be due to the high accumulation of Cd in the root parenchyma cells, which play a functional role in enhancing resistance to the radical flow of Cd. It was in the line of previous findings where a high accumulation of Cd often recorded in roots than shoots^7^. While Si application in our experiment significantly alleviated Cd toxicity by further increasing roots diameter and volume (Fig. 1). In our study, low and moderate concentrations of Si (1 and 3 mM) traps more level of Cd in root through vacuolar sequestrations, which were reflected by a further increase in root average diameter and volume (Fig. 1). The same results were recorded by Greger et al.^34^, where Si traps more concentration of Cd in root cell walls.

Si plays a central role in plant development, particularly under unfavorable conditions like heavy metal and acidic stresses^47,51,52^. Previous findings have shown different effects on plant growth and yield with various levels of Si concentrations^52^. The recorded data of the present findings indicate that Si accumulation in the roots of wheat seedlings grown under Si treaded acidic nutrient solution is entirely associated with root fresh and dry weights (Fig. 1). The application of Si (1 or 3 mM) in acidic nutrient solution boosted up root growth-related parameters, and it improved Si root accumulation in wheat seedlings against the corresponding acidic pH single treatment (Fig. 1). Though, the incorporation of Si 3 mM (high concentration) upregulated root morphology and physiological traits compared with Si 1 mM (low concentration) because Si deposition in wheat roots increases with the increase of Si amount in growth solution and also increases the roots capability to absorb Si^53^. The present study also supports the conclusion of previous findings (Fig. 1 and Table 2). It has been reported by previous researchers that silica deposition in nature is strongly correlated with many chemicals, environmental factors, including, pH, silica concentrations, temperature, as well as the presence of other polymers, small compounds in a nutrient solution, and different cations and anions^54^. Acidic pH could release the toxicity perceived in roots of wheat seedlings treated with a high Si concentration (3 Mm) (Fig. 1), which might be due to a fact of an acidic pH prevents Si installation^54^. Though, this hypothesis still needs to be more deliberate.

The present study demonstrated that the root physiology and morphology treated with Si in the acidic nutrient solution is strongly associated with the root biomass (Fig. 1). Root development and yield are strongly affected by essential nutrient uptake and accumulation^45,55^. The vital nutrients like; N, P, K, Ca, Mg, and Zn influenced strongly on plant growth. K is a crucial element to the various metabolic reaction and organic plant structuring due to its significant contribution to enzyme activation, protein synthesis, and photosynthesis reaction. Ca contributes to maintaining cell wall configuration and membrane performance^56^. Moreover, Ca deals with the physical and biological responses of plants by transduction of stress signals under various stresses^57^. The negative impact of acidic pH on seed emergent, growth, and root morphology and physiology could be improved by the incorporation of Ca. The previous researchers have shown that on behalf of Ca requirements, and different plants often show different levels of tolerance to acidic pH^58^. As an important component of chlorophyll and an enzyme cofactor, Mg plays a crucial role in carbon fixation and photosynthetic intensity^59^. Micronutrients, like Zn, play an important role in the organic plant structure, biochemical reactions, and metabolic activity of plants^60^. The results of previous findings have demonstrated that macro and microelements can affect plant growth and development to varying extents^61,62^. Therefore, the balanced amounts of nutrient elements are essential for adequate growth and survival of wheat plants under worse conditions of Cd and acidic pH. Several studies have shown that acidic pH inhibits the uptake and utilization of essential nutrients and thereby affects plant optimum growth^46,47^. Our study herein supports the conclusion of previous findings, but toxicities triggered by Cd and acidic pH were strongly encountered by the incorporation of Si. Specifically, treatment with Cd (200 µM) along with acidic pH (5) significantly diminished the root biomass (FW and DW) of wheat plants, significantly hindered the uptake of N, P, K, Ca, Mg, and Si, and visibly improved the concentrations of Zn and Cd (Table 2). The addition of Si (1 or 3 mM) in acidic nutrient solution significantly amplified the wheat root biomass (F.W. and D.W.) and reassured any severe rises or falls (Fig. 1). Our findings demonstrated that Si in the wheat roots or Si treatment significantly disturbed the root uptake and utilization of essential nutrients and well-maintained the relative equilibrium of the mineral elements in roots (Table 2). Similar conclusions have been drawn in previous findings^51,63^, and changed concentrations of mineral nutrients might establish a tool for the hindering or upregulating root growth^10,61^.

Reactive oxygen species (ROS) in terms of hydrogen peroxide (H_2_O_2_) and peroxidation of membrane lipids in terms of MDA contents caused by the environmental stresses can be caused the irrevocable cellular injury through their intensive oxidative behavior^9^, which endorse variations in normal morphological and physiological structure of roots that enhance resistance^64^. In the current study, herein, a parallel response was recorded in the roots of wheat seedlings grown under low or high concentrations of Cd (50 or 200 µM) in the acidic growing media (Table 1). Moreover, the addition of Si in Cd contaminated acidic nutrient solution remarkably reduced the contents of H_2_O_2_ and MDA in roots of wheat seedlings (Table 1). Previous studies have demonstrated that the incorporation of Si reduced the H_2_O_2_ and MDA contents in plants under hostile conditions of stresses^31^, demonstrating that Si ameliorates oxidative burst tempted by acidic pH as well as Cd toxicity.

Although the overproduction of ROS can cause cell loss, it is needed to regulate ROS contents to a safe level in plants. Plants have enzymatic (superoxide dismutase; SOD, catalase; CAT, and peroxidase; POD) and non-enzymatic (glutathione; GHS, and ascorbic acid; AsA) antioxidants and osmoprotectants (total soluble sugar; TSS and proline) to remove ROS contents to confer resistance to stress^10,46^. In enzymatic antioxidants, SOD is more affective in demolishing ROS^65^. Our experimental results demonstrated that SOD activity was remarkably improved in the roots of wheat seedlings grown in low or high concentrations of Cd (50 or 200 µM) in an acidic nutrient solution, the incorporation of Si in Cd-contaminated acidic nutrient solution further improved SOD activity than that of the parallel Cd single treatment (Table 1), demonstrating improved alleviation of oxidative stress. Parallel results have revealed in earlier researchers that Si treatment demolishes Cd stress by improving the functioning of antioxidants in both roots and shoots of plants^31,66^. CAT, as a crucial antioxidant enzyme, participates in the deduction of harmful peroxides in root cells through the breakdown of H_2_O_2_ compounds into H_2_O and O_2_. A significant increase in CAT contents in roots of wheat seedlings under low or high concentrations of Cd in the acidic nutrient solution (50 or 200 µM) was reported in the recent findings (Table 1), and an equivalent improvement was stated in previous results^31,67^. However, Si moderate or high concentration (1 or 3 mM) significantly reversed the adverse effects of Cd toxicity by further increasing in CAT contents in the roots of wheat plants under Cd exposure as compared with corresponding Cd single treatment (Table 1), representing that Si improves the antioxidant or scavenger contents and thereby decreases free radicals of oxidants, as was formerly reported^45,46,63^. POD also involves demolishing H_2_O_2_ in below-ground parts of various plant species^10,47^. In the current study, POD contents were improved in the roots of wheat plants grown under low or high concentrations of Cd (50 or 200 µM) in acidic nutrient solution, the addition of Si moderate or severe concentrations (1 or 3 mM) in Cd-contaminated acidic nutrient solution further increased POD activity (Table 1).

In the non-enzymatic antioxidative ROS-scavenging mechanism, AsA, as a frontline of defense in the intervention with exterior oxidant injury^68^, plays a central role in demolishing H_2_O_2_^69^. In our study, herein, AsA flow in roots of wheat seedlings was enhanced with the treatment of low or high level of Cd (50 or 200 µM) in an acidic nutrient solution, the addition of Si moderate or high concentrations (1 or 3 mM) in Cd-contaminated acidic nutrient solution further enhanced AsA flow in roots of wheat plants, indicated Si demolishing effect to Cd and acidic pH morphological impacts (Table 1). GSH displays a central role in cell tolerance to metal stresses by regulating redox imbalance and by minimizing the free ion cellular concentration above and below-ground parts of plants^70^. Our experimental data demonstrated that GSH activity was increased in the roots of wheat plants grown under Cd low or high concentrations (50 or 200 µM), the addition of Si moderate or high concentrations ( 1 or 3 mM) in Cd-contaminated acidic nutrient solution further increased GSH activity (Table 1), indicated Si role in regulating redox imbalance.

Osmoprotectants, in terms of total soluble sugar (TSS) and proline contents, maintain the homeostasis of ROS in both root and shoot cells, consequently protects the plants from various stresses^71^. TSS as an important osmoprotectant provides membrane protection by regulating the osmotic adjustment and by scavenging toxic ROS under various abiotic stresses^72^. Proline plays an essential role in stress tolerance by stabilizing the redox status of plant cells^73^. Moreover, proline is considered as an antioxidant to scavenge ROS and reserve the intercellular pool, a key redox buffer for cells^74^. In our study, herein, the activity of TSS and proline was increased in the roots of wheat plants treated with low and high concentrations of Cd (50 and 200 µM) along with acidic pH, the addition of Si in Cd-contaminated acidic nutrient solution further increased the activity of TSS and proline, indicated the Si role to preserver the intercellular pool to stabilize the redox status of cells (Table 1).

It has been recommended in prior findings that Si is an effective quasi-essential element that could participate actively in several morphological, physiological, and structural reactions in higher plants grown under biotic and abiotic environmental strains^31,75^, and the results of our study supported this assumption. In summary, on the one hand, Si incorporated with safe concentrations (1 and 3 mM) significantly improves the morphology and physiology of the roots of wheat seedlings, increases the activities of CAT, SOD, POD, AsA, GSH, TSS, and proline in roots of wheat plants, affects the uptake and consumption of mineral nutrients, and alleviates the acidic pH toxicity. On the other hand, Si addition also improves the combined toxicities of acidic pH and Cd concentrations in roots of wheat seedlings by demolishing ROS contents and by increasing the activities of enzymatic and non-enzymatic antioxidants and osmoprotectant contents.

## Supplementary Information

Supplementary Information 1.

## Data Availability

The data that support the findings of this study are presented in this manuscript. Raw data of enzymatic study and plasma mass spectroscopy in this research will be available on request after acceptance of the paper.
