# Supplementary material for: Alleviatory effects of Silicon on the morphology, physiology, and antioxidative mechanisms of wheat (Triticum aestivum L.) roots under cadmium stress in acidic nutrient solutions
Source: Sci Rep. 2021 Jan 21;11:1958. doi: 10.1038/s41598-020-80808-x (PMC7820580; doi:10.1038/s41598-020-80808-x)
Supplement: Supplementary file 1 — Supplementary Information 1. [file 41598_2020_80808_MOESM1_ESM.docx]

**Alleviatory effects of Silicon on the morphology, physiology and antioxidative mechanisms of wheat (*Triticum aestivum* L.) roots under cadmium stress in acidic nutrient solution**

Shafeeq-ur-Rahman^1,2*,^ Qi Xuebin^1,2^, Zhijuan Zhao^1,2^, Muhammad Imtiaz^3^, Faisal Mehmood^1,2^, Lu Hongfei^1,2^, Babar Hussain^4^, Muhammad Nadeem Ashrif^4^,

^1^Farmland Irrigation Research Institute, Chinese Academy of Agricultural Sciences, Xinxiang, 453003, China.

^2^Key Laboratory of High-efficient and Safe Utilization of Agriculture Water Resources of CAAS, Xinxiang, 453003, China.

^3^Soil and Environmental Biotechnology Division, National Institute for Biotechnology and Genetic Engineering (NIBGE).

^4^Institute of Agricultural Resources and Regional Planning, Chinese Academy of Agricultural Sciences.

*Corresponding author at Farmland Irrigation Research Institute, Chinese Academy of Agriculture Sciences, Xinxiang, Henan 453003, P.R. China.

Tel: +86-153-33738321

E-mail address: [malikshafeeq1559@gmail.com](mailto:malikshafeeq1559@gmail.com) (Shafeeq-ur-Rahman

**1. Recipe of Hoagland’s solution**

The Hoagland's solution had composition (mg L-1): (NH4)2SO4 48.2, MgSO4 65.9, K2SO4 15.9, KNO3 18.5, Ca (NO3)2 59.9, KH2PO4 24.8, Fe citrate 6.8, MnCl2.4H2O 0.9, ZnSO4.7H2O 0.11, CuSO4.5H2O 0.04, H3BO3 2.9, H2MoO4 0.01.

**2. Recipe of Si treatment**

Silicon (Si) as a silica nanoparticle prepared from sodium silicate. A measured amount of sodium silicate put into the double amount of boiling deionized distilled water in a petri dish for ten minutes. For increasing its solubility, we added a small amount of KOH and further heated for 10 minutes. Continue stirring with a long-handled spoon and at the end added 2 to 3 drops of H_2_O_2_. The solution removed from heat stove and allowed it to cool at room temperature. Cooled solution transferred into a plastic bottle and sealed it up. From the prepared stock solution, we made subsolution of 3mM per liter and applied as SiF3 and SiR3. Both treatments Si and Cd were applied after 65 days of transplantation. The twelve treatments were arranged factorially in a randomized complete block design with three replications per treatments. A total number of pots were 36, and each pot contained 15 plants.

**3. Determination of nutrient elements in plant tissues**

“The contents of micronutrients (nitrogen: N, phosphorus: P, and potassium: K), macronutrients (calcium: Ca, magnesium: Mg, and zinc: Zn were assessed by inductively coupled plasma mass spectroscopy (ICP-MS, Agilent, and 7700 X, USA) after being oven-dried by following the methods of previous researchers (Fırat et al. 2017). Total nitrogen was measured in root samples by the method of (Brookes et al., 1985) with some modifications. Weighted 0.3 grams of root dry samples in digestion tubes with 4 ml of sulphuric acid were put on the curved stem small funnel into the digestion furnace and initiated to dissolve. The temperature was set to 220 ̊C for 2h and boiled till brown-yellow endpoint. First, samples were heated at 380 °C and removed these digestion tubes from the digestion furnace. Added 20 drops of hydrogen peroxide (H_2_O_2_) and root samples were placed on a hot plate and continue to heat again. This cycle was repeated 2-5 times to clear the digestion solution. At every cycle, the amount of H_2_O_2_ was decreased. The amount of H_2_O_2_ added to the blank was consistent with the maximum number of samples. After that, a few amounts of multiple flushing methods were transferred to the 100 ml volumetric flask. In the end, total nitrogen was determined in a solution using flow analyzer-3 of brand BRAN+LUEBBE, and potassium was determined in a solution using flame photometer FP6410”(Shafeeq ur et al. 2020).

4. **Determination of Cd and Si concentration in roots**

“After 120 days of the experiment, wheat plants were harvested and washed thoroughly with tap water, distilled water, and then with double distilled water. Plant samples were separated into roots and shoots and dried at 70 °C in the oven for 48 h, and ground into powder. Weighed 0.2 grams of dried root samples were placed into a microwave digestion tube. Added 10 ml of nitric acid and covered every sample with a lid. Later, it was put into the microwave digestion instrument and started to dissolve after the selection of an appropriate program. After completion of digestion, tubes were removed and put the digestion solution into the PTFE digestion cup with a small number of repeated flushing. Digestion cups were placed on a hot plate at 220 °C temperature until the removal of 2-3 ml liquid. In the end, the digestion fluid was transferred into the 50 ml volumetric flask and made its volume up to 50 ml with double distilled water. Digestion solution used to measure Cd concentrations in root samples by using an atomic absorption spectrophotometer (AAS) model AA-6300 SHIMADZU.

Si concentration in roots was measured according to the modified autoclave-induced digestion method by Elliott and Snyder (1991). In brief, dried plant samples were dissolved with 3 mL of 50% NaOH solution at 121 °C for 2 h. Afterward, 30 mL 20% acetic acid, 10 mL ammonium molybdate (54 g·L^−1^, pH 7.0), 5 mL 20% tartaric acid, and 1 mL reductant were added to 1 mL of the samples. After 30 min, Si concentration of the samples was measured at 650 nm on a TU-1901 UV-Vis spectrophotometer (TU-1901, Beijing Purkinje General Instrument Co., Ltd., Beijing, China)” (Shafeeq ur et al. 2020).

.
